# Associations between chronic conditions and death in hospital among adults (aged 20+ years) during first acute care hospitalizations with a confirmed or suspected COVID-19 diagnosis in Canada

**DOI:** 10.1371/journal.pone.0280050

**Published:** 2023-01-04

**Authors:** Dianne Zakaria, Samina Aziz, Sharon Bartholomew, Su-Bin Park, Cynthia Robitaille, Murray Weeks

**Affiliations:** Centre for Surveillance and Applied Research, Health Promotion and Chronic Disease Prevention Branch, Public Health Agency of Canada, Ottawa, Ontario, Canada; Johns Hopkins University Bloomberg School of Public Health, UNITED STATES

## Abstract

**Purpose:**

We aimed to quantify life course-specific associations between death in hospital and 30 chronic conditions, and comorbidity among them, in adults (aged 20+ years) during their first acute care hospitalization with a confirmed or suspected COVID-19 diagnosis in Canada.

**Methods:**

We identified 35,519 first acute care hospitalizations with a confirmed or suspected COVID-19 diagnosis in the Discharge Abstract Database as of March 31, 2021. For each of five life-course age groups (20–34, 35–49, 50–64, 65–79, and 80+ years), we used multivariable logistic regression to examine associations between death in hospital and 30 chronic conditions, comorbidity, period of admission, and pregnant status, after adjusting for sex and age.

**Results:**

About 20.9% of hospitalized patients with COVID-19 died in hospital. Conditions most strongly associated with in-hospital death varied across the life course. Chronic liver disease, other nervous system disorders, and obesity were statistically significantly associated (α = 0.05) with in-hospital death in the 20–34 to 65–79 year age groups, but the magnitude of the associations decreased as age increased. Stroke (aOR = 5.24, 95% CI: 2.63, 9.83) and other inflammatory rheumatic diseases (aOR = 4.37, 95% CI: 1.64, 10.26) were significantly associated with in-hospital death among 35 to 49 year olds only. Among 50+ year olds, more chronic conditions were significantly associated with in-hospital death, but the magnitude of the associations were generally weaker except for Down syndrome in the 50 to 64 (aOR = 8.49, 95% CI: 4.28, 16.28) and 65 to 79 year age groups (aOR = 5.19, 95% CI: 1.44, 20.91). Associations between comorbidity and death also attenuated with age. Among 20 to 34 year olds, the likelihood of death was 19 times greater (aOR = 18.69, 95% CI: 7.69, 48.24) in patients with three or more conditions compared to patients with none of the conditions, while for 80+ year olds the likelihood of death was two times greater (aOR = 2.04, 95% CI: 1.70, 2.45) for patients with six or more conditions compared to patients with none of the conditions.

**Conclusion:**

Conditions most strongly associated with in-hospital death among hospitalized adults with COVID-19 vary across the life course, and the impact of chronic conditions and comorbidity attenuate with age.

## Introduction

The cumulative burden of coronavirus disease 2019 (COVID-19) in Canada is substantial. To date, nearly 4.4 million infections, confirmed by polymerase chain reaction, have been reported to the Public Health Agency of Canada [1]. Although a minority of cases are hospitalized, detailed examination of their characteristics and outcomes is important because they are costly, characterized by substantial morbidity, and strain health care systems [1–4]. Several countries have published national level data documenting the characteristics of hospitalized patients with COVID-19 and factors associated with adverse outcomes [5–7]. Age, sex, and a growing number of chronic conditions have been associated with poorer outcomes, but little research conducted on hospitalized patients with COVID-19 has explored how these factors interact [8, 9]. Knowing more about these interactions can inform clinical practice, public health strategies, future research, and our understanding of the virus’ mechanisms of action. One Canadian study characterized patients hospitalized with a COVID-19 diagnosis, but it was limited to a small geographic region during the first wave of the pandemic, contained relatively few patients (N = 972), and did not examine associations between specific chronic conditions and adverse outcomes [10]. Considering the paucity of studies exploring interactions both internationally and nationally, a comprehensive examination of Canadian data is warranted. We aimed to quantify life course-specific associations between in-hospital death and 30 chronic conditions, and comorbidity among them, in adults (aged 20+ years) during their first acute care hospitalization with a confirmed or suspected COVID-19 diagnosis in Canada.

## Methods

### Data source

We obtained data on all acute care hospital discharges between April 1, 2009 and March 31, 2021 for Canada, excluding Quebec, from the Discharge Abstract Database (DAD) [11]. We uniquely defined people by combining year of birth, province/territory issuing the health card number, and anonymized health card number. Year of birth was included to distinguish mothers from their newborns who may be temporarily assigned their mother’s health card number.

### Defining the COVID-19 cohort

The admission date for a person’s first acute care hospitalization with COVID-19 was based on their earliest DAD record with a COVID-19 diagnosis (S1 Table) in any of the 25 diagnosis fields. Since DAD records for an individual can be nested, overlapping or sequential in time resulting in continuous hospitalization days across multiple facilities, we combined all such records to capture a person’s episode of care. A calendar day without acute care hospitalization ended an episode. Only DAD acute care records belonging to males or females with known person identifiers, admission and discharge dates and times, and age at admission contributed to episodes. In the DAD files used in our analyses, the percentage of records missing information on any of these variables never exceeded 1.1%. Adults aged 20 years and older at admission with a confirmed (International Statistical Classification of Diseases and Related Health Problems, 10^th^ revision, Canada code U07.1) or suspected (U07.2) COVID-19 diagnosis in their first COVID-19 episode of care were retained for further analysis. We excluded patients who were less than 20 years of age at admission, who had a sex other than male or female (less than five patients), or who had unknown person identifiers, admission/discharge dates/times, or age. We included suspected COVID-19 cases because testing capacity was limited early in the pandemic, potentially resulting in decisions as to who should be tested, and lab results may not have been available at the time of patient discharge. In support of this statement, our data indicate that suspected COVID-19 cases accounted for 9.9% of cases prior to September 1, 2020 (wave one) but only 1.7% of cases between September 1, 2020 and March 31, 2021 (wave two).

### Defining health conditions, comorbidity and the primary outcome

We defined health conditions considered higher risk for more severe COVID-19 [12, 13] using several sources (S2 Table) [14–19]. We searched all 25 diagnosis fields on all DAD records in the episode to determine health condition status. As we were interested in conditions present prior to the episode, we excluded diagnoses arising during the episode by using the diagnosis type field. To obtain a more complete picture of a person’s health status, we linked COVID-19 episodes of care to historical DAD records from the previous 10 fiscal years and searched all diagnosis fields without regard for diagnosis type. For health conditions that are not necessarily permanent (cancers and obesity), only the previous two fiscal years of DAD records were used while pregnant status was based solely on diagnoses within the episode. Our approach for cancer is based on research indicating cancer diagnoses documented within the past two years in administrative health data were more consistently associated with adverse outcomes than older cancer diagnoses [20]. Two measures of comorbidity were examined: the Charlson comorbidity index, a prognostic indicator of mortality (S3 Table) [18, 21, 22]; and the number of chronic conditions documented for a person. To calculate the Charlson comorbidity index, we used the same approach to search DAD records for relevant conditions. Our primary outcome of interest during the COVID-19 episode of care was in-hospital death.

### Analyses

Since the prevalence of conditions varied substantially across the life course and preliminary analyses often identified interactions between sex, age, and chronic conditions, all analyses were performed by life-course age groups (20–34, 35–49, 50–64, 65–79, and 80+ years). Life-course age groups are characterized by life events, social roles, and chronic conditions typically experienced during the age group (e.g., education, childbearing, careers, retirement, advanced age). Descriptive statistics included proportions for categorical data and medians with interquartile range for continuous data. Multivariable logistic regression was used to examine associations between in-hospital death and all 30 chronic conditions of interest, period of COVID-19 admission, and pregnancy status, after adjusting for sex and age. Period of COVID-19 admission (Sep 1, 2020 or later vs earlier, i.e., second vs first wave) was included because research has demonstrated improvements in patient outcomes over time [6, 23–26]. Pregnancy status was included as an adjustment variable to address the large number of hospitalizations related to deliveries in younger age groups. For comparison with these life course-specific models, we also provide the initial model with all ages combined. Comorbidity was examined in separate models to prevent masking of condition-specific associations.

To ensure more parsimonious, numerically stable models that were more generalizable [27], we used stepwise selection [28], with an alpha level of 0.05 to enter and remain in the model, to identify variables significantly associated with in-hospital death after adjusting for age and sex. If the Hosmer-Lemeshow goodness-of-fit test indicated poor model fit, the continuous age variable was examined to ensure linearity [27], and interactions involving sex were assessed by testing product terms involving sex and each of the retained variables at an alpha level of 0.05. We found categorical age did not perform better than continuous age, and none of the identified interactions were qualitative in nature (i.e., positive association in one sex and negative association in the other sex). Thus, age was always modelled as a continuous variable and models were not split by sex.

For brevity, when communicating results for chronic conditions, we do not consistently state the comparison group, by default the complement. We also use *significant* interchangeably with *statistically significant*.

To prevent disclosure, we suppress estimates based on counts of 1 to 4 and additional estimates, as needed, to prevent residual disclosure through differencing. We used SAS Enterprise Guide 7.1 for all analyses and prepared this manuscript using guidelines for reporting of studies using routinely collected health data [29].

### Ethics statement

Our study was exempt from research ethics board review under article 2.2 of the Tri-Council Policy Statement: Ethical Conduct for Research Involving Humans [30]. Pursuant to article 2.2, research does not require research ethics board review when it relies exclusively on information that is publicly available through a mechanism set out by legislation or regulation and that is protected by law. Exemption is based on the presence of a custodian/steward, designated in accordance with access to information and privacy legislation, who protects privacy and proprietary interests associated with the information. Our study involved analysis of previously collected anonymized/de-identified (i.e., no direct identifiers) administrative and clinical data; did not involve linkage to additional data sources; did not include direct follow-up or contact of patients; and adhered to the data providers terms and conditions of access, use, and dissemination, including privacy and confidentiality guidelines to prevent disclosure.

## Results

**Fig 1:**
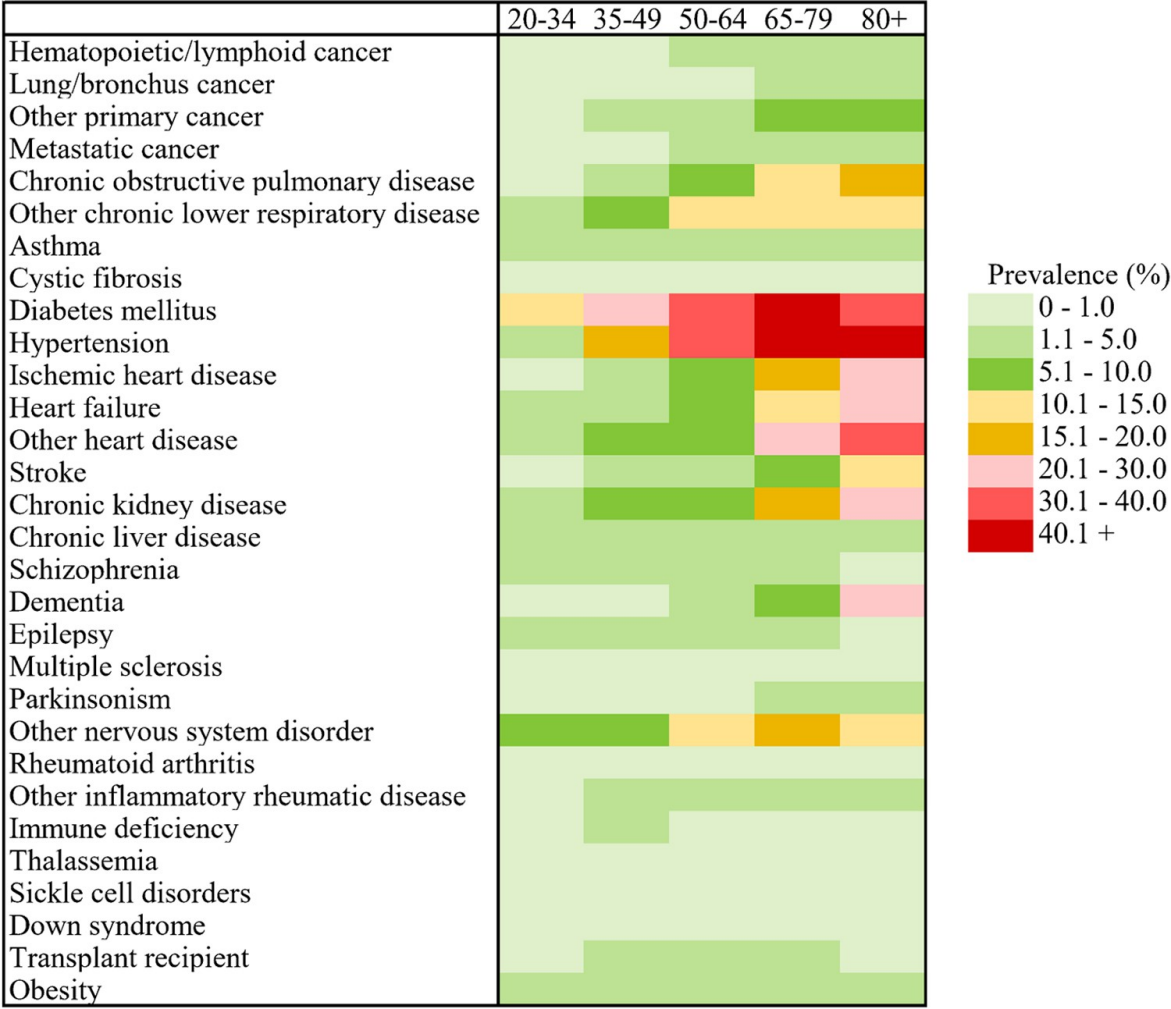
Prevalence (%) of chronic conditions among adults (aged 20+ years) during first acute care hospitalizations with a confirmed or suspected COVID-19 diagnosis in Canada by life-course age group. Includes acute care hospitalizations ending by March 31, 2021 in Canada, excluding Quebec. For detailed estimates see Table 2. COVID-19 = coronavirus disease 2019.

As a whole, our study population of 35,519 adult patients were slightly more likely to be male (54.0%); had a median age at admission of 69.0 years (IQR: 54.0 to 81.0); consisted predominantly of confirmed COVID-19 cases (96.6%) admitted after the first wave (79.1%); and were primarily admitted from the community (72.9%) (Table 1). Substantial morbidity was experienced: 22.5% were admitted to an intensive care unit, 12.6% received invasive mechanical ventilation, and 20.9% died in hospital. The most prevalent chronic conditions were hypertension (41.9%) and diabetes (37.8%), and 75.5% had at least one of the 30 chronic conditions (Table 2, Fig 1). Using up to 10 fiscal years of historical data to identify chronic conditions had a substantial impact on prevalence. For 12 of the 30 conditions, prevalence more than doubled (S4 Table).

**Table 1:** Characteristics of first acute care hospitalizations for adults (aged 20+ years) with a confirmed or suspected COVID-19 diagnosis in Canada by life-course age group.

	20+ N* = 35519		20 to 34 N = 2476		35 to 49 N = 4189		50 to 64 N = 8303		65 to 79 N = 10632		80+ N = 9919	
Characteristics	N^Ϯ^	Estimate^‡^	N	Estimate	N	Estimate	N	Estimate	N	Estimate	N	Estimate
Male	19197	54.0	976	39.4	2340	55.9	5049	60.8	6173	58.1	4659	47.0
Age at admission in years^§^	35519	69.0 (54.0,81.0)	2476	29.0 (26.0,32.0)	4189	43.0 (39.0,47.0)	8303	58.0 (54.0,61.0)	10632	72.0 (69.0,76.0)	9919	86.0 (83.0,90.0)
Infection status												
confirmed	34297	96.6	2325	93.9	4040	96.4	8020	96.6	10315	97.0	9597	96.8
suspected	1222	3.4	151	6.1	149	3.6	283	3.4	317	3.0	322	3.2
Admitted Sep 1, 2020 or later	28113	79.1	2067	83.5	3377	80.6	6444	77.6	8452	79.5	7773	78.4
Admitted from												
acute inpatient care/emergency department	1421	4.0	80	3.2	141	3.4	316	3.8	473	4.4	411	4.1
inpatient complex continuing care/long-term care (24 hour nursing care)/palliative care	2510	7.1	-^¶^	-	-	-	191	2.3	792	7.4	1493	15.1
nursing stations/day surgery/other health facility/ambulatory care clinic/community mental health and addiction services/community based clinic	2024	5.7	175	7.1	300	7.2	530	6.4	633	6.0	386	3.9
group living/supportive housing/transitional housing/home care program	3286	9.3	84	3.4	108	2.6	303	3.6	711	6.7	2080	21.0
admitted from community	25908	72.9	2098	84.7	3550	84.7	6886	82.9	7929	74.6	5445	54.9
other	370	1.0	-	-	-	-	77	0.9	94	0.9	104	1.0
Pregnant	869	2.4	663	26.8	206	4.9	0	0.0	0	0.0	0	0.0
Discharge status												
home with/without support	21222	59.7	2079	84.0	3471	82.9	6208	74.8	6216	58.5	3248	32.7
transfer to another hospital/emergency/ambulatory care	2842	8.0	90	3.6	203	4.8	664	8.0	922	8.7	963	9.7
transfer to residential care/group/supportive living	3414	9.6	106	4.3	162	3.9	441	5.3	920	8.7	1785	18.0
died in facility	7435	20.9	53	2.1	161	3.8	822	9.9	2509	23.6	3890	39.2
other	606	1.7	148	6.0	192	4.6	168	2.0	65	0.6	33	0.3
Episode length in days^§^^¥^	35519	9.0 (4.0,19.0)	2476	4.0 (2.0,7.0)	4189	5.0 (3.0,10.0)	8303	8.0 (4.0,16.0)	10632	11.0 (5.0,22.0)	9919	12.0 (6.0,26.0)
Admitted to intensive care unit	7991	22.5	364	14.7	976	23.3	2515	30.3	3044	28.6	1092	11.0
Received invasive mechanical ventilation	4486	12.6	196	7.9	523	12.5	1511	18.2	1830	17.2	426	4.3

Note: Includes acute care hospitalizations ending by March 31, 2021 in Canada, excluding Quebec. COVID-19 = coronavirus disease 2019.

*Number of individuals in age group.

ϮNumber of individuals with characteristic.

‡Percentage unless otherwise indicated.

§Median and interquartile range.

¶For confidentiality, estimates based on 1 to 4 people having a characteristic are suppressed. Additional estimates may be suppressed to prevent residual disclosure through differencing.

¥An episode of care begins on the admission date of the earliest acute care hospitalization with a COVID-19 diagnosis and continues until the first calendar day without acute care hospitalization. Consequently, episodes of care can include transfers across acute care hospitals.

**Table 2:** Chronic condition prevalence and comorbidity among adults (aged 20+ years) during first acute care hospitalizations with a confirmed or suspected COVID-19 diagnosis in Canada by life-course age group.

	20+N* = 35519		20 to 34 N = 2476		35 to 49 N = 4189		50 to 64 N = 8303		65 to 79 N = 10632		80+N = 9919	
	N^Ϯ^	Estimate^‡^	N	Estimate	N	Estimate	N	Estimate	N	Estimate	N	Estimate
Chronic conditions												
hematopoietic/lymphoid cancer	580	1.6	12	0.5	21	0.5	122	1.5	251	2.4	174	1.8
lung/bronchus cancer	324	0.9	-^¶^	-	-	-	48	0.6	153	1.4	119	1.2
other primary cancer	1692	4.8	17	0.7	66	1.6	300	3.6	645	6.1	664	6.7
metastatic cancer	816	2.3	11	0.4	34	0.8	188	2.3	350	3.3	233	2.3
chronic obstructive pulmonary disease	3730	10.5	9	0.4	56	1.3	564	6.8	1559	14.7	1542	15.5
other chronic lower respiratory disease	4032	11.4	109	4.4	335	8.0	877	10.6	1476	13.9	1235	12.5
asthma	1307	3.7	115	4.6	197	4.7	333	4.0	381	3.6	281	2.8
cystic fibrosis	12	0.0	-	-	-	-	-	-	-	-	-	-
diabetes mellitus	13439	37.8	277	11.2	1018	24.3	3192	38.4	5159	48.5	3793	38.2
hypertension	14883	41.9	112	4.5	679	16.2	2691	32.4	5359	50.4	6042	60.9
ischemic heart disease	5418	15.3	14	0.6	120	2.9	805	9.7	2122	20.0	2357	23.8
heart failure	4717	13.3	35	1.4	131	3.1	563	6.8	1565	14.7	2423	24.4
other heart disease	6907	19.4	100	4.0	216	5.2	823	9.9	2269	21.3	3499	35.3
stroke	2549	7.2	15	0.6	73	1.7	365	4.4	898	8.4	1198	12.1
chronic kidney disease	5409	15.2	68	2.7	259	6.2	820	9.9	1951	18.4	2311	23.3
chronic liver disease	1038	2.9	61	2.5	147	3.5	339	4.1	349	3.3	142	1.4
schizophrenia	593	1.7	70	2.8	95	2.3	165	2.0	212	2.0	51	0.5
dementia	4061	11.4	-	-	-	-	150	1.8	1059	10.0	2841	28.6
epilepsy	664	1.9	52	2.1	78	1.9	210	2.5	223	2.1	101	1.0
multiple sclerosis	144	0.4	-	-	-	-	52	0.6	46	0.4	24	0.2
parkinsonism	665	1.9	-	-	-	-	50	0.6	274	2.6	338	3.4
other nervous system disorder	4386	12.3	173	7.0	385	9.2	1003	12.1	1601	15.1	1224	12.3
rheumatoid arthritis	234	0.7	-	-	-	-	41	0.5	106	1.0	75	0.8
other inflammatory rheumatic disease	380	1.1	13	0.5	45	1.1	92	1.1	115	1.1	115	1.2
immune deficiency	243	0.7	14	0.6	60	1.4	84	1.0	70	0.7	15	0.2
thalassemia	60	0.2	10	0.4	7	0.2	17	0.2	13	0.1	13	0.1
sickle cell disorders	51	0.1	14	0.6	19	0.5	-	-	8	0.1	-	-
Down syndrome	77	0.2	12	0.5	14	0.3	41	0.5	10	0.1	0	0.0
transplant recipient	393	1.1	22	0.9	50	1.2	142	1.7	155	1.5	24	0.2
obesity	1228	3.5	119	4.8	192	4.6	361	4.3	442	4.2	114	1.1
Any of the 30 chronic conditions	26831	75.5	845	34.1	2152	51.4	5754	69.3	9025	84.9	9055	91.3
Number of chronic conditions^§^	35519	2.0 (1.0,3.0)	2476	0.0 (0.0,1.0)	4189	1.0 (0.0,2.0)	8303	1.0 (0.0,3.0)	10632	2.0 (1.0,4.0)	9919	3.0 (2.0,4.0)
Charlson comorbidity index^§^	35519	1.0 (0.0,2.0)	2476	0.0 (0.0,0.0)	4189	0.0 (0.0,1.0)	8303	0.0 (0.0,1.0)	10632	1.0 (0.0,3.0)	9919	2.0 (1.0,3.0)

Note: Includes acute care hospitalizations ending by March 31, 2021 in Canada, excluding Quebec. Number of chronic conditions has an upper limit of 30 and the Charlson comorbidity index has an upper limit of 24. COVID-19 = coronavirus disease 2019.

*Number of individuals in age group.

ϮNumber of individuals with characteristic.

‡Percentage unless otherwise indicated.

¶For confidentiality, estimates based on 1 to 4 people having a characteristic are suppressed. Additional estimates may be suppressed to prevent residual disclosure through differencing.

§Median and interquartile range.

### Factors associated with adverse outcomes

#### Sex and age

Final multivariable models indicated the likelihood of death among 20 to 34 year olds was 3.5 times greater (aOR = 3.54, 95% CI: 1.91, 6.80) in males compared to females while the association was not statistically significant among 35 to 49 year olds (aOR = 1.21, 95% CI: 0.86, 1.71) and ranged from 1.33 (95% CI: 1.14, 1.56) in 50 to 64 year olds to 1.51 (95% CI: 1.39, 1.65) in 80+ year olds (Table 3, S5 Table).

**Table 3:** Associations between demographic, temporal and health characteristics and death in hospital among adults aged 20+ years during first acute care hospitalizations with a confirmed or suspected COVID-19 diagnosis in Canada by life-course age group.

	20+ (D* = 7435/N^†^ = 35519)	20 to 34 (D = 53/N = 2476)	35 to 49 (D = 161/N = 4189)	50 to 64 (D = 822/N = 8303)	65 to 79 (D = 2509/N = 10632)	80+ (D = 3890/N = 9919)
	aOR (95% CI)	aOR (95% CI)	aOR (95% CI)	aOR (95% CI)	aOR (95% CI)	aOR (95% CI)
Male (vs female)	1.43 (1.35,1.52)	3.54 (1.91,6.80)	1.21 (0.86,1.71)	1.33 (1.14,1.56)	1.50 (1.37,1.66)	1.51 (1.39,1.65)
Age (per year)	1.06 (1.06,1.06)	0.93 (0.87,1.00)	1.05 (1.01,1.09)	1.07 (1.05,1.09)	1.05 (1.03,1.06)	1.05 (1.04,1.06)
Admitted Sep 1, 2020 or later (vs earlier)	0.88 (0.82,0.94)					0.86 (0.78,0.95)
Pregnant (vs not pregnant)	-^‡^					
Chronic conditions^§^						
hematopoietic/lymphoid cancer	1.71 (1.42,2.06)		-	2.57 (1.59,4.02)	1.69 (1.28,2.21)	
lung/bronchus cancer	1.78 (1.39,2.27)	-		2.81 (1.38,5.60)	2.15 (1.50,3.07)	
other primary cancer				1.72 (1.15,2.51)		
metastatic cancer	2.39 (2.04,2.81)		6.78 (2.49,16.26)	2.60 (1.63,4.15)	2.00 (1.56,2.54)	1.93 (1.48,2.53)
chronic obstructive pulmonary disease	1.23 (1.14,1.34)			1.67 (1.30,2.12)	1.26 (1.11,1.43)	1.13 (1.01,1.27)
other chronic lower respiratory disease	1.30 (1.19,1.41)			1.55 (1.25,1.91)	1.23 (1.08,1.40)	1.28 (1.13,1.45)
asthma						
cystic fibrosis						
diabetes mellitus	1.15 (1.08,1.22)			1.34 (1.14,1.57)		1.14 (1.04,1.24)
hypertension						
ischemic heart disease	1.10 (1.02,1.18)					
heart failure	1.20 (1.11,1.30)		2.28 (1.22,4.04)		1.25 (1.10,1.43)	1.27 (1.15,1.41)
other heart disease						
stroke			5.24 (2.63,9.83)			
chronic kidney disease	1.45 (1.35,1.56)			2.23 (1.82,2.73)	1.61 (1.43,1.80)	1.31 (1.19,1.45)
chronic liver disease	2.66 (2.29,3.08)	21.05 (9.80,44.08)	6.68 (4.09,10.68)	3.28 (2.51,4.27)	2.05 (1.63,2.57)	
schizophrenia					1.54 (1.14,2.08)	
dementia	1.37 (1.27,1.48)				1.46 (1.26,1.68)	1.35 (1.23,1.48)
epilepsy						
multiple sclerosis						
parkinsonism	1.31 (1.10,1.55)					
other nervous system disorder	1.22 (1.13,1.32)	7.06 (3.62,13.42)	3.01 (2.01,4.44)	1.29 (1.05,1.57)	1.18 (1.04,1.34)	
rheumatoid arthritis	1.50 (1.12,2.01)				1.82 (1.20,2.74)	
other inflammatory rheumatic disease			4.37 (1.64,10.26)			
immune deficiency						
thalassemia		-				
sickle cell disorders						
Down syndrome	5.05 (2.94,8.43)			8.49 (4.28,16.28)	5.19 (1.44,20.91)	
transplant recipient	1.44 (1.12,1.83)					
obesity	1.48 (1.27,1.72)	5.42 (2.05,12.62)	3.08 (1.72,5.24)	1.40 (1.02,1.90)	1.38 (1.11,1.72)	

Note: Includes acute care hospitalizations ending by March 31, 2021 in Canada, excluding Quebec. All final multivariable models are adjusted for sex and age, irrespective of statistical significance, but other retained variables must be significant at an alpha of 0.05. aOR = adjusted odds ratio, CI = confidence interval, COVID-19 = coronavirus disease 2019, D = in-hospital deaths, N = number of individuals, NA = not applicable, uOR = unadjusted odds ratio.

*Number of in-hospital deaths in the age group.

ϮNumber of adults in the age group.

‡For confidentiality, estimates based on counts of 1 to 4 are suppressed.

§The reference group are those without the chronic condition.

Among 20 to 34 year olds, the likelihood of death significantly decreased by 7% per additional year of age (aOR = 0.93, 95% CI: 0.87,1.00; p = 0.0456). Conversely, the likelihood of death significantly increased 5% to 7% per additional year of age for adults aged 35 to 49 (aOR = 1.05, 95% CI: 1.01, 1.09), 50 to 64 (aOR = 1.07, 95% CI: 1.05,1.09), 65 to 79 (aOR = 1.05, 95% CI: 1.03,1.06), and 80+ years (aOR = 1.05, 95% CI: 1.04,1.06).

#### Period of admission and pregnancy status

Among 20 to 79 year olds, admission after the first wave was not significantly associated with a lower likelihood of death (Table 3). Conversely, among 80+ year olds, admission after the first wave was significantly associated with a lower likelihood of death (aOR = 0.86, 95% CI: 0.78, 0.95). Post hoc analyses among 80+ year olds indicated those admitted after the first wave were significantly less likely to originate from continuing or long term care (12.6% vs 23.9%, Fisher’s exact two-sided p<0.0001), and were significantly less likely to have hypertension (60.2% vs 63.6%, Fisher’s exact two-sided p = 0.0047) and dementia (27.4% vs 33.0%, Fisher’s exact two-sided p<0.0001) (S6 Table).

Although pregnancy status was selected into the final overall model which included adults aged 20 and older, the number of deaths were too few to present results. However, post hoc analyses for females aged 20 to 49 years indicated that pregnant females were significantly less likely to have many of the chronic conditions compared to non-pregnant females (S7 Table). Overall, among 20 to 34 year olds, non-pregnant females were 3.7 times more likely than pregnant females to have at least one chronic condition (44.0% vs 11.9%, Fisher’s exact two-sided p<0.0001); and, among 35 to 49 year olds, non-pregnant females were 4.2 times more likely than pregnant females to have at least one chronic condition (54.9% vs 13.1%, Fisher’s exact two-sided p<0.0001).

#### Chronic conditions by life course

Among 20 to 34 year olds, significant associations were noted between death and chronic liver disease (aOR = 21.05, 95% CI: 9.80, 44.08), other nervous system disorder (aOR = 7.06, 95% CI: 3.62, 13.42), and obesity (aOR = 5.42, 95% CI: 2.05, 12.62) (Table 3). Similarly, 35 to 49 year olds demonstrated significant associations between death and chronic liver disease (aOR = 6.68, 95% CI: 4.09, 10.68), other nervous system disorder (aOR = 3.01, 95% CI: 2.01,4.44), and obesity (aOR = 3.08, 95% CI: 1.72, 5.24). Additionally, significant associations between death and metastatic cancer (aOR = 6.78, 95% CI: 2.49, 16.26), heart failure (aOR = 2.28, 95% CI: 1.22, 4.04), stroke (aOR = 5.24, 95% CI: 2.63, 9.83), and other inflammatory rheumatic disease (aOR = 4.37, 95% CI: 1.64, 10.26) existed.

Among 50 to 64 year olds, significant associations existed between death and hematopoietic/lymphoid cancer (aOR = 2.57, 95% CI: 1.59, 4.02), lung/bronchus cancer (aOR = 2.81, 95% CI: 1.38, 5.60), other primary cancer (aOR = 1.72, 95% CI: 1.15, 2.51), metastatic cancer (aOR = 2.60, 95% CI: 1.63, 4.15), chronic obstructive pulmonary disease (aOR = 1.67, 95% CI: 1.30, 2.12), other chronic lower respiratory disease (aOR = 1.55, 95% CI: 1.25, 1.91), diabetes mellitus (aOR = 1.34, 95% CI: 1.14,1.57), chronic kidney disease (aOR = 2.23, 95% CI: 1.82, 2.73), chronic liver disease (aOR = 3.28, 95% CI: 2.51,4.27), other nervous system disorder (aOR = 1.29, 95% CI: 1.05, 1.57), Down syndrome (aOR = 8.49, 95% CI: 4.28, 16.28), and obesity (aOR = 1.40, 95% CI: 1.02, 1.90). Except for other primary cancer and diabetes mellitus, all these chronic conditions were also significantly associated with death among 65 to 79 year olds, but the magnitude of associations tended to be smaller. Some additional chronic conditions were significantly associated with death among 65 to 79 year olds, but not 50 to 64 year olds: heart failure (aOR = 1.25, 95% CI: 1.10, 1.43), schizophrenia (aOR = 1.54, 95% CI: 1.14, 2.08), dementia (aOR = 1.46, 95% CI: 1.26, 1.68), and rheumatoid arthritis (aOR = 1.82, 95% CI: 1.20, 2.74). For 80+ year olds, fewer chronic conditions were significantly associated with death compared to adults aged 50 to 64 and 65 to 79 years, and the magnitude of the associations were generally weak.

Post hoc analyses, limited to age groups having patients with Down syndrome (ages 20 to 79 years), indicated that people with Down syndrome were, on average, younger than those without the condition (50.8 vs 58.4 years, t-test two-sided p<0.0001). A comparison of chronic condition prevalence across the two groups showed people with Down syndrome were less likely to have diabetes mellitus (20.8% vs 37.7%, Fisher’s exact two-side p = 0.0020), hypertension (14.3% vs 34.6%, Fisher’s exact two-side p<0.0001), and ischemic heart disease (0.0% vs 12.0%, Fisher’s exact two-side p = 0.0001); and more likely to have other chronic lower respiratory disease (20.8% vs 10.9%, Fisher’s exact two-side p = 0.0097), dementia (31.2% vs 4.7%, Fisher’s exact two-side p<0.0001), and epilepsy (10.4% vs 2.2%, Fisher’s exact two-side p = 0.0003) (S8 Table). Overall, patients with Down syndrome were just as likely to have one or more of the remaining chronic conditions compared to those without Down syndrome (69.3% vs 71.4%, Fisher’s exact two-sided p = 0.8046).

#### Comorbidity by life course

The impact of comorbidity varied by life-course age group (Table 4). Relative to adults with none of the conditions, having three or more conditions was significantly associated with a 19-fold increase in the likelihood of death among 20 to 34 year olds (aOR = 18.69, 95% CI: 7.69, 48.24) and a 14-fold increase among 35 to 49 year olds (aOR = 13.95, 95% CI: 8.35, 24.49). Among 50 to 64 year olds, having six or more conditions was associated with a 7-fold increase in the likelihood of death (aOR = 7.36, 95% CI: 5.47, 9.90) relative to those with none of the conditions. For adults aged 65 to 79 years and 80+ years, having six or more conditions increased the likelihood of death by a factor of 3.68 (95% CI: 3.04, 4.46) and 2.04 (95% CI: 1.70, 2.45), respectively, relative to those with none of the conditions. Overall, the strength of association between death and condition count decreased with advancing age (see S9 Table for findings using the Charlson comorbidity index).

**Table 4:** Association between chronic condition count and death in hospital among adults aged 20+ years during first acute care hospitalization with a confirmed or suspected COVID-19 diagnosis in Canada by life-course age group.

Number of chronic conditions	20 to 34 (D = 53/N = 2476)	35 to 49 (D = 161/N = 4189)	50 to 64 (D = 822/N = 8303)	65 to 79 (D = 2509/N = 10632)	80+ (D = 3890/N = 9919)
	aOR (95% CI)	aOR (95% CI)	aOR (95% CI)	aOR (95% CI)	aOR (95% CI)
1 vs 0	6.95 (3.09,17.08)	4.09 (2.37,7.37)	1.69 (1.30,2.20)	1.48 (1.23,1.79)	1.04 (0.87,1.25)
2 vs 0	14.31 (6.01,36.45)	9.26 (5.35,16.67)	2.82 (2.18,3.68)	1.98 (1.65,2.39)	1.22 (1.03,1.46)
3+ vs 0	18.69 (7.69,48.24)	13.95 (8.35,24.49)	4.48 (3.42,5.88)	2.54 (2.11,3.06)	1.32 (1.11,1.57)
4 vs 0			4.26 (3.12,5.81)	2.76 (2.27,3.36)	1.73 (1.45,2.07)
5 vs 0			7.47 (5.45,10.23)	3.33 (2.70,4.10)	1.77 (1.47,2.14)
6+ vs 0			7.36 (5.47,9.90)	3.68 (3.04,4.46)	2.04 (1.70,2.45)

Note: Includes acute care hospitalizations ending by March 31, 2021 in Canada, excluding Quebec. All final multivariable models are adjusted for sex and age, irrespective of statistical significance, and pregnancy status and period of admission when significant at an alpha of 0.05. For age groups 20 to 34 and 35 to 49 years, the highest category is 3 or more chronic conditions; for all other age groups, the highest category is 6 or more chronic conditions. aOR = adjusted odds ratio, CI = confidence interval, COVID-19 = coronavirus disease 2019, D = in-hospital deaths in the age group, N = number of adults in the age group.

### Sensitivity analysis

We conducted post hoc sensitivity analyses to examine the impact of limiting our multivariable modelling to confirmed COVID-19 patients (S10 Table). Overall, findings were generally consistent with those that included suspected cases (Table 3). Some additional chronic conditions were selected into the final model for those aged 50 to 64 years (parkinsonism), 65 to 79 years (other heart disease, parkinsonism), and 80+ years (hematopoietic/lymphoid cancer) (S10 Table). In addition, period of admission was selected into the final model for patients aged 50 to 64 years (aOR = 0.83, 95% CI: 0.69, 1.00) and 65 to 79 years (aOR = 0.84, 95% CI: 0.75, 0.95) indicating the likelihood of in-hospital death was lower for those admitted Sep 1, 2020 or later compared to those admitted earlier (S10 Table). Post hoc analyses examining the prevalence of chronic conditions among 50 to 79 year olds indicated suspected cases were generally more likely than confirmed cases to have a condition when significant differences existed (S11 Table). Consistent with this finding, suspected cases were more likely than confirmed cases to have at least one of the chronic conditions examined (83.3% vs 77.9%, Fisher’s exact two-sided p = 0.0013). However, suspected cases were less likely than confirmed cases to be admitted to an intensive care unit (18.8% vs 29.7%, Fisher’s exact two-sided p<0.0001), receive invasive mechanical ventilation (7.8% vs 18.0%, Fisher’s exact two-sided p<0.0001), and die in the hospital (11.3% vs 17.8%, Fisher’s exact two-sided p<0.0001). Similar to findings for all age groups combined, suspected cases accounted for a greater proportion of admissions in wave one than wave two among 50 to 79 year olds (8.9% vs 1.6%, Fisher’s exact two-sided p<0.0001).

## Discussion

Using a large health administrative database, we found that adults suffer substantial morbidity during first acute care hospitalizations with a confirmed or suspected COVID-19 diagnosis: 22.5% are admitted to an intensive care unit, 12.6% receive invasive mechanical ventilation, and 20.9% die in hospital (Table 1). We also found numerous significant associations between chronic conditions and in-hospital death. Our findings are generally consistent with previous research [5–7, 10, 19, 20, 31–42] and are not surprising, as many of the factors identified (e.g., male sex, advancing age, chronic conditions, accrual of chronic conditions) are associated with an increased risk of death from infectious disease in general [43]. Nonetheless, our stratified analyses revealed differences across the life course. With respect to sex, our findings for those aged 50+ years are in line with studies of hospitalized cohorts in the United Kingdom and United States where measures of association with death range from 1.18 to 1.46 [5–7, 35, 37]. However, among 20 to 34 year olds, the likelihood of death was 3.5 times greater in males than females, whereas the association between sex and death was not statistically significant in 35 to 49 year olds (Table 3). Pregnant females, who accounted for 26.8% of the youngest age group (Table 1), likely contributed to this effect. Compared to non-pregnant females, pregnant females were significantly less likely to have chronic conditions (S7 Table) and likely differ in ways not captured by the model. Further, since pregnant females generally deliver in a hospital, we may be capturing females who incidentally test positive for SARS-CoV-2 but have mild COVID-19. The greater likelihood of severe COVID-19 in males compared to females has been hypothesized to result from greater angiotensin converting enzyme-2 expression in males [44, 45], and immunological advantages of the female sex attributed to genetic and hormonal factors [46]. Gender differences, not quantifiable in the DAD, may also contribute.

Consistent with our findings, others have found that the strength of associations between chronic conditions, including obesity, and severe COVID-19 can attenuate with age [8, 9, 20, 47–51], and that strong associations exist between death and liver disease, cardiovascular disease and cancer among confirmed cases younger than age 50 years [48]. Similarly, we found that the strength of association between comorbidity and the likelihood of death decreased across the life course. Although others have found that greater comorbidity is associated with more severe COVID-19 [34, 38, 52], few have demonstrated that associations are strongest among the youngest [48]. Several factors, related to age, may be contributing to these findings. First, background mortality increases with age so the potential relative impact of a specific condition decreases with age. Second, for each condition examined, both those with and those without the condition of interest may have additional conditions. Consequently, the increasing number of chronic conditions with age could have attenuated condition-specific effects. Last, we examined 30 conditions, not all conditions, and older age groups are more likely to have other conditions not captured in our model. Despite these general trends, we found strong associations between Down syndrome and in-hospital death in adults aged 50 to 64 years and 65 to 79 years (Table 3) that are consistent with other research [20, 40], and likely reflect the comorbidity and lower life expectancy experienced by people with Down syndrome [53, 54]. Our post hoc analyses revealed that, despite being younger on average, people with Down syndrome were just as likely to have one or more of the remaining chronic conditions compared to those without Down syndrome.

The lower likelihood of death in the second wave compared to the first among 80+ year olds may reflect changes in the characteristics of this age group over time that were not fully captured by the model. For example, our post hoc analyses indicated that those admitted after the first wave were less likely to originate from continuing or long term care. When limiting our model to confirmed cases in the sensitivity analysis, period of admission was also selected into the final model for patients aged 50 to 64 years and 65 to 79 years (S10 Table). However, post hoc drill down analyses suggested that the suspected cases may have been less severely affected. Considering suspected cases accounted for a greater proportion of admissions in wave one relative to wave two, their removal may have biased the comparison of waves by creating a more severely affected wave one patient population. These findings suggest testing may have been limited to more severely affected patients at the start of the pandemic when testing capacity was limited.

### Limitations

Several limitations should be acknowledged when interpreting the results of our work. First, chronic conditions of interest may not have been consistently captured in acute care hospital data. Among the chronic conditions we examined, only diabetes mellitus must be coded when documented. For the other conditions, mandatory reporting is based on criteria like the condition increasing a patient’s length of stay, or the condition co-occurring with other specific conditions that have been coded [55]. To minimize this limitation, we used up to 10 fiscal years of historical data to identify conditions. Nonetheless, prevalence estimates for some of the chronic conditions remained below general population estimates (e.g., chronic obstructive pulmonary disease, asthma, rheumatoid arthritis and obesity) (S4 Table). Under-ascertainment of conditions could result in biasing measures of association towards the null because reference groups are not free of the disease. Second, our study is limited to patients discharged from hospital. Systematic differences between patients that remain in hospital and those discharged could bias our results. Third, in the absence of a control group, we were unable to determine if the existence of chronic conditions were a unique risk for people with COVID-19, or generally a risk for in-hospital death irrespective of infection status. Fourth, death after discharge from hospital is not captured resulting in an underestimation of the burden of COVID-19. Fifth, the lower prevalence of chronic conditions and fewer adverse outcomes in younger age groups resulted in lower power to detect smaller effect sizes relative to older age groups. Last, although we excluded diagnoses arising during the COVID-19 episode, some conditions may have developed as a result of the infection prior to the acute care hospital admission. To minimize these limitations, future research should use more integrated administrative, laboratory, and surveillance data to minimize under-ascertainment, incorporate socioeconomic factors, include appropriate control groups, standardize follow-up, and establish temporality of chronic conditions, SARS-CoV-2 infection and adverse outcomes. Internationally accepted chronic disease definitions based on the International Statistical Classification of Diseases and Related Health Problems would improve comparability.

## Conclusions

Chronic conditions most strongly associated with in-hospital death among hospitalized adults with COVID-19 vary across the life course, and the impact of chronic conditions and comorbidity attenuate with age. We recommend stratifying analyses by age to prevent masking of associations by the more numerous older patients, and acknowledging the importance of comorbidity, particularly among younger patients. Our detailed methodological approach and life course-specific findings provide useful information for a wide audience. Clinicians can use our findings to provide more personalized patient management; researchers can use our detailed methods and results to inform future COVID-19 research; and public health professionals can use our findings to refine communication, testing, shielding, and vaccination strategies.

## Supporting information

S1 Table ICD-10-CA codes for COVID-19. (DOCX)

S2 Table Health condition definitions. (DOCX)

S3 Table Charlson comorbidity index. (DOCX)

S4 Table Prevalence estimates (%) for chronic conditions among adults (aged 20+ years) in Canada by data source and life course age group. (DOCX)

S5 Table Associations between demographic, temporal and health characteristics and death in hospital among adults aged 20+ years during first acute care hospitalizations with a confirmed or suspected COVID-19 diagnosis in Canada by life-course age group. (XLSX)

S6 Table Prevalence of chronic conditions among adults aged 80+ years during first acute care hospitalizations with a confirmed or suspected COVID-19 diagnosis in Canada by period of admission. (DOCX)

S7 Table Prevalence of chronic conditions among females aged 20 to 49 years during first acute care hospitalizations with a confirmed or suspected COVID-19 diagnosis in Canada by pregnancy status. (DOCX)

S8 Table Prevalence of chronic conditions among adults aged 20 to 79 years during first acute care hospitalizations with a confirmed or suspected COVID-19 diagnosis in Canada by Down syndrome status. (DOCX)

S9 Table Associations between Charlson comorbidity index and death in hospital among adults (aged 20+ years) during first acute care hospitalizations with a confirmed or suspected COVID-19 diagnosis in Canada by life-course age group. (DOCX)

S10 Table Associations between demographic, temporal and health characteristics and death in hospital among adults (aged 20+ years) during first acute care hospitalizations with a confirmed COVID-19 diagnosis in Canada by life-course age group. (XLSX)

S11 Table Prevalence of chronic conditions among adults aged 50 to 79 years during first acute care hospitalizations with a confirmed or suspected COVID-19 diagnosis in Canada by COVID-19 status. (DOCX)
