# Supplementary material for: Associations between chronic conditions and death in hospital among adults (aged 20+ years) during first acute care hospitalizations with a confirmed or suspected COVID-19 diagnosis in Canada
Source: PLoS One. 2023 Jan 4;18(1):e0280050. doi: 10.1371/journal.pone.0280050 (PMC9812329; doi:10.1371/journal.pone.0280050)
Supplement: S1 Table — (DOCX) [file pone.0280050.s001.docx]

| S1 Table. ICD-10-CA^1^ codes for COVID-19 | |
| --- | --- |
| Code | Use |
| U07.1 | COVID-19 has been confirmed by laboratory results. |
| U07.2 | COVID-19 is suspected (i.e., diagnosed clinically or epidemiologically) but lab results are inconclusive or not available, or testing is not performed. |
| U07.3 | Multisystem inflammatory syndrome associated with COVID-19. |
| U07.4 | Used to classify a post COVID-19 condition. The ICD-10-CA codes for the specific condition(s) or symptom(s) associated with a past COVID-19 infection are assigned a prefix code of 7 to identify them as being associated with the past COVID-19 infection. |
| U07.5 | Personal history of COVID-19. |
| Note: COVID-19 = coronavirus disease 2019, ICD-10-CA = International Statistical Classification of Diseases and Related Health Problems, 10th revision, Canada. | |

References:

1. Canadian Institute for Health Information [Internet]. COVID-19 data collection and coding direction [cited 2020 Dec 11]. Available from: <https://www.cihi.ca/en/covid-19-resources/covid-19-data-collection-and-coding-direction>.
