# Supplementary material for: Associations between chronic conditions and death in hospital among adults (aged 20+ years) during first acute care hospitalizations with a confirmed or suspected COVID-19 diagnosis in Canada
Source: PLoS One. 2023 Jan 4;18(1):e0280050. doi: 10.1371/journal.pone.0280050 (PMC9812329; doi:10.1371/journal.pone.0280050)
Supplement: S2 Table — (DOCX) [file pone.0280050.s002.docx]

| S2 Table. Health condition definitions | |
| --- | --- |
| Health condition | ICD-10-CA Codes |
| Pregnant | Z33 Pregnant state, incidental  Z37 Outcome of delivery  O00-O99 Pregnancy, childbirth and the puerperium, excluding codes that have a 6^th^ digit of 4 (indicates postpartum condition or complication). Additional codes excluded from O00-O99: complications following abortion and ectopic and molar pregnancy (O08) when used without a code from O00 to O05; sequelae of complication of pregnancy, childbirth and the puerperium (O94); death from any obstetric cause occurring more than 42 days but less than one year after delivery (O96); and, death from sequelae of obstetric causes (O97). |
| Hematopoietic/lymphoid cancer | C81-C96 Malignant neoplasms, stated or presumed to be primary, of lymphoid, haematopoietic and related tissue |
| Lung/bronchus cancer | C34 Malignant neoplasm of bronchus and lung |
| Other primary invasive cancers | C00-C76, C97 excluding liver cell carcinoma (C22.0), which is included in chronic liver disease, and lung/bronchus cancer (C34) |
| Metastatic cancer^1^ | C77 Secondary and unspecified malignant neoplasm of lymph nodes  C78 Secondary malignant neoplasm of respiratory and digestive organs  C79 Secondary malignant neoplasm of other and unspecified sites  C80 Malignant neoplasm, without specification of site |
| Chronic obstructive lung disease^2^ | J41 Simple and mucopurulent chronic bronchitis  J42 Unspecified chronic bronchitis  J43 Emphysema  J44 Other chronic obstructive pulmonary disease |
| Other chronic lower respiratory disease^3*^ | G47.3 Sleep apnoea  J40 Bronchitis, not specified as acute or chronic  J47 Bronchiectasis  J60-J70 Lung diseases due to external agents  J84 Other interstitial pulmonary diseases  J98.0 Diseases of bronchus, not elsewhere classified  J98.2 Interstitial emphysema  J98.4 Other disorders of lung  J98.6 Disorders of diaphragm  J98.8 Other specified respiratory disorders  J98.9 Respiratory disorder unspecified  J99 Respiratory disorders in diseases classified elsewhere |
| Asthma^2ϯ^ | J45 Asthma |
| Cystic fibrosis | E84 Cystic fibrosis |
| Diabetes mellitus^2^ | E10 Insulin-dependent diabetes mellitus  E11 Non-insulin-dependent diabetes mellitus  E13 Other specified diabetes mellitus  E14 Unspecified diabetes mellitus  The above diagnoses are not acknowledged if gestational diabetes (O24.8) is documented on the same DAD record |
| Hypertension^2^ | I10 Essential (primary) hypertension  I11 Hypertensive heart disease  I12 Hypertensive renal disease  I13 Hypertensive heart and renal disease  I15 Secondary hypertension  The above diagnoses are not acknowledged if gestational hypertension (O13), pre-eclampsia (O14), eclampsia (O15), or unspecified maternal hypertension (O16) are documented on the same DAD record |
|  |  |
| Ischemic heart disease^2^ | I20 Angina pectoris  I21 Acute myocardial infarction  I22 Subsequent myocardial infarction  I23 Certain current complications following acute myocardial infarction  I24 Other acute ischaemic heart diseases  I25 Chronic ischaemic heart disease |
| Heart failure^2^ | I50 Heart failure |
| Other heart disease^3*^ | I01 Rheumatic fever with heart involvement  I05-I09 Chronic rheumatic heart diseases  I27 Other pulmonary heart disease  I28 Other diseases of pulmonary vessels  I31 Other diseases of pericardium  I34 Nonrheumatic mitral valve disorders  I35 Nonrheumatic aortic valve disorders  I36 Nonrheumatic tricuspid valve disorders  I37 Pulmonary valve disorders  I42 Cardiomyopathy  I43 Cardiomyopathy in diseases classified elsewhere  I44 Atrioventricular and left bundle-branch block  I45 Other conduction disorders  I47 Paroxysmal tachycardia  I48 Atrial fibrillation and flutter  I49 Other cardiac arrhythmias  151.0-I51.2 and I51.5-I51.9 Complications and ill-defined descriptions of heart disease  I52 Other heart disorders in diseases classified elsewhere |
| Stroke^2^ | G08 Intracranial and intraspinal phlebitis and thrombophlebitis  G45 Transient cerebral ischaemic attacks and related syndromes excluding G45.4  H34.0 Transient retinal artery occlusion  H34.1 Central retinal artery occlusion  I60 Subarachnoid haemorrhage  I61 Intracerebral haemorrhage  I63 Cerebral infarction  I64 Stroke, not specified as haemorrhage or infarction  I67.6 Nonpyogenic thrombosis of intracranial venous system |
| Chronic kidney disease^3*^ | N08.3 Glomerular disorders in diabetes mellitus  N18 Chronic kidney disease  Z49 Care involving dialysis  Z94.0 Kidney transplant status  Z99.2 Dependence on renal dialysis |
| Chronic liver disease including hepatocellular carcinoma^3*^ | C22.0 Liver cell carcinoma  I85.0 Oesophageal varices with bleeding  I98.3 Oesophageal varices with bleeding in diseases classified elsewhere  K70.2 Alcoholic fibrosis and sclerosis of liver  K70.3 Alcoholic cirrhosis of liver  K70.4 Alcoholic hepatic failure  K71.7 Toxic liver disease with fibrosis and cirrhosis of liver  K72.0 Acute and subacute hepatic failure  K72.1 Chronic hepatic failure  K72.9 Hepatic failure, unspecified  K73 Chronic hepatitis, not elsewhere classified  K74.0 Hepatic fibrosis  K74.1 Hepatic sclerosis  K74.2 Hepatic fibrosis with hepatic sclerosis  K74.3 Primary biliary cirrhosis  K74.4 Secondary biliary cirrhosis  K74.5 Biliary cirrhosis, unspecified  K74.6 Other and unspecified cirrhosis of liver  K76.7 Hepatorenal syndrome  R18 Ascites |
| Schizophrenia^2^ | F20 Schizophrenia  F21 Schizotypal disorder  F23 Acute and transient psychotic disorders  F25 Schizoaffective disorders |
| Dementia^2^ | F00 Dementia in Alzheimer disease  F01 Vascular dementia  F02 Dementia in other diseases classified elsewhere  F03 Unspecified dementia  G30 Alzheimer disease |
| Epilepsy^2^ | G40 Epilepsy |
| Multiple sclerosis^2^ | G35 Multiple sclerosis |
| Parkinsonism^2^ | F02.3 Dementia in Parkinson disease  G20 Parkinson disease  G21 Secondary parkinsonism  G22 Parkinsonism in diseases classified elsewhere |
| Other nervous system disorder^3*^ | G10-G14 Systemic atrophies primarily affecting the central nervous system  G23 Other degenerative diseases of basal ganglia  G24 Dystonia  G25 Other extrapyramidal and movement disorders  G26 Extrapyramidal and movement disorders in diseases classified elsewhere  G31 Other degenerative diseases of nervous system, not elsewhere classified  G32 Other degenerative disorders of nervous system in diseases classified elsewhere  G36 Other acute disseminated demyelination  G37 Other demyelinating diseases of central nervous system  G41 Status epilepticus  G43 Migraine  G44 Other headache syndromes  G45.4 Transient global amnesia  G46 Vascular syndromes of brain in cerebrovascular diseases  G47 Sleep disorders (excluding G47.3 which is included in other chronic lower respiratory disease)  G50-G59 Nerve, nerve root and plexus disorders  G60-G64 Polyneuropathies and other disorders of the peripheral nervous system  G70-G73 Diseases of myoneural junction and muscle  G80-G83 Cerebral palsy and other paralytic syndromes  G90-G99 Other disorders of the nervous system |
| Rheumatoid arthritis^2^ | M05 Seropositive rheumatoid arthritis  M06 Other rheumatoid arthritis  Must be diagnosed 730 days prior to COVID-19 admission date |
| Other inflammatory rheumatic disease^4^ | I77.6 Arteritis, unspecified  L40.5, M07.0-M07.3 Psoriatic arthritis  L95 Vasculitis limited to skin, not elsewhere classified  M08.0, M08.2-M08.9 Juvenile rheumatoid arthritis  M08.1 Juvenile ankylosing spondylitis  M30.0 Polyarteritis nodosa  M32 Systemic lupus erythematosus  M33 Dermatopolymyositis  M34.0, M34.1, M34.8, M34.9 Systemic sclerosis  M35.0 Sicca syndrome [Sjögren]  M35.1 Other overlap syndromes  M35.3 Polymyalgia rheumatica  M45 Ankylosing spondylitis |
| Immune deficiency^3*^ | B24 Human immunodeficiency virus disease  O987 HIV disease complicating pregnancy  D80-D89 Certain disorders involving the immune mechanism |
| Thalassemia | D56 Thalassemia |
| Sickle cell disorders | D57 Sickle cell disorders |
| Down syndrome | Q90 Down syndrome |
| Transplant recipient | T86 Failure and rejection of transplanted organs and tissues  Z94 Transplanted organ and tissue status |
| Obesity | E66 Obesity |
| Note: DAD = Discharge Abstract Database, HIV = human immunodeficiency virus, ICD-10-CA = International Statistical Classification of Diseases and Related Health Problems, 10^th^ revision, Canada.  *Modifications were made to chronic kidney disease, other nervous system disease, and chronic liver disease definitions based on feedback from Canadian Institute for Health Information classification specialists. O987 was added to immune deficiency based on the Charlson comorbidity index (Appendix 3). Other chronic lower respiratory disease was modified to remove lung conditions that may result from COVID-19 (e.g. J80 adult respiratory distress syndrome). Other heart disease was modified to remove heart conditions that may result from COVID-19 (e.g. I26 pulmonary embolism, I40 acute myocarditis).  ϯThe ICD-10 code for status asthmaticus (J46) is not a valid ICD-10-CA code. The presence of status asthmaticus in combination with asthma is captured in the ICD-10-CA code for asthma (J45). | |

References:

1. Quan H, Sundararajan V, Halfon P, Fong A, Burnand B, Luthi JC, et al. Coding algorithms for defining comorbidities in ICD-9-CM and ICD-10 administrative data. Med Care 2005;43(11):1130-9.
2. Government of Canada [Internet]. Canadian Chronic Disease Surveillance System (CCDSS) [cited 2020 Dec 11]. Available from: <https://health-infobase.canada.ca/ccdss/data-tool/>.
3. McGurnaghan SJ, Weir A, Bishop J, Kennedy S, Blackbourn LAK, McAllister DA, et al. Risks of and risk factors for COVID-19 disease in people with diabetes: a cohort study of the total population of Scotland. Lancet Diabetes Endocrinol 2021;9(2):82-93.
4. Shin YH, Shin JI, Moon YS, Jin HY, Kim SY, Yang JM, et al. Autoimmune inflammatory rheumatic diseases and COVID-19 outcomes in South Korea: a nationwide cohort study. Lancet Rheumatol 2021;3(10):e698-e706.
