# Supplementary material for: Associations between chronic conditions and death in hospital among adults (aged 20+ years) during first acute care hospitalizations with a confirmed or suspected COVID-19 diagnosis in Canada
Source: PLoS One. 2023 Jan 4;18(1):e0280050. doi: 10.1371/journal.pone.0280050 (PMC9812329; doi:10.1371/journal.pone.0280050)
Supplement: S3 Table — (DOCX) [file pone.0280050.s003.docx]

| S3 Table. Charlson comorbidity Index^1-3^ | | |
| --- | --- | --- |
| Conditions | ICD-10-CA codes | Weight |
| Congestive heart failure | I099, I255, I420, I425, I426, I427, I428, I429, I43, I50, P290 | 2 |
| Dementia | F00^*^, F01, F02, F03, F051, G30, G311 | 2 |
| Chronic pulmonary disease | I278, I279, J40, J41, J42, J43, J44, J45, J47, J60, J61, J62, J63, J64, J65, J66, J67, J684, J701, J703 | 1 |
| Rheumatologic diseases | M05, M06, M315, M32, M33, M34, M351, M353, M360 | 1 |
| Diabetes with chronic complications | E102, E103, E104, E105, E107, E112, E113, E114, E115, E117, E132, E133, E134, E135, E137, E142, E143, E144, E145, E147 | 1 |
| Hemiplegia or paraplegia | G041, G114, G801, G802, G81, G82, G830, G831, G832, G833, G834, G839 | 2 |
| Renal disease | N032, N033, N034, N035, N036, N037, N052, N053, N054, N055, N056, N057, N18, N19, N250, Z490, Z491, Z492, Z940^*^, Z992^*^ | 1 |
| Mild liver disease | B18, K700, K701, K702, K703, K709, K713, K714, K715, K717, K73, K74, K760, K762, K763, K764, K768, K769, Z944^*^ | 2 |
| Moderate or severe liver disease | I850, I859, I864, K704, K711, K721, K729, K765, K766, K767 | 4 |
| AIDS/HIV | B24, O987^ϯ^ | 4 |
| Any malignancy, including lymphoma and leukemia | C0, C1, C20, C21, C22, C23, C24, C25, C26, C30, C31, C32, C33, C34, C37, C38, C39, C40, C41, C43, C45, C46, C47, C48, C49, C50, C51, C52, C53, C54, C55, C56, C57, C58, C6, C70, C71, C72, C73, C74, C75, C76, C81, C82, C83, C84, C85, C88, C90, C91, C92, C93, C94, C95, C96, C97 | 2 |
| Metastatic solid tumour | C77, C78, C79, C80 | 6 |
| Note: Adapted from the Canadian Institute for Health Information (2020).^1^ Comorbid conditions and weights are based on work performed by Quan et al (2011, 2005).^2,3^ When calculating the Charlson comorbidity index, people are classified as either having or not having each condition. People who satisfy the definition of mild liver disease and moderate or severe liver disease are classified as having moderate or severe liver disease. Similarly, people who satisfy the definition of any malignancy and metastatic solid tumour are classified as having metastatic solid tumour. The overall Charlson comorbidity index is obtained by summing the weighted conditions. The following ICD-10 codes used by Quan et al (2005) do not appear in this table because they are not valid ICD-10-CA codes: B20 to B22, E122 to E125, I110, I120, I130, I131, I132, and J46. AIDS/HIV = acquired immune deficiency syndrome/human immunodeficiency virus, CIHI = Canadian Institute for Health Information, ICD-10-CA = International Statistical Classification of Diseases and Related Health Problems, 10th revision, Canada.  *Do not appear in CIHI algorithm. Included based on work performed by Quan et al (2005).^3^  ϮDoes not appear in Quan et al (2005).^3^ Included based on CIHI algorithm.^1^ | | |

References:

1. Canadian Institute for Health Information. Indicator Library: General Methodology Notes — Clinical Indicators, November 2020. Ottawa, ON: CIHI; 2020.
2. Quan H, Li B, Couris C, Fushimi K, Graham P, Hider P, et al. Updating and validating the Charlson comorbidity index and score for risk adjustment in hospital discharge abstracts using data from 6 countries. Am J Epidemiol 2011;173:676–82.
3. Quan H, Sundararajan V, Halfon P, Fong A, Burnand B, Luthi JC, et al. Coding algorithms for defining comorbidities in ICD-9-CM and ICD-10 administrative data. Med Care 2005;43(11):1130-9.
