# Supplementary material for: Associations between chronic conditions and death in hospital among adults (aged 20+ years) during first acute care hospitalizations with a confirmed or suspected COVID-19 diagnosis in Canada
Source: PLoS One. 2023 Jan 4;18(1):e0280050. doi: 10.1371/journal.pone.0280050 (PMC9812329; doi:10.1371/journal.pone.0280050)
Supplement: S4 Table — (DOCX) [file pone.0280050.s004.docx]

| S4 Table. Prevalence estimates (%) for chronic conditions among adults (aged 20+ years) in Canada by data source and life course age group | | | | | | | |
| --- | --- | --- | --- | --- | --- | --- | --- |
| Chronic condition | Data source | 20+ N^*^=35519 | 20 to 34 N=2476 | 35 to 49 N=4189 | 50 to 64 N=8303 | 65 to 79 N=10632 | 80+ N=9919 |
| Hematopoietic/lymphoid cancer | E^Ϯ^ | 1.28 | 0.44 | 0.45 | 1.17 | 1.86 | 1.32 |
|  | E+H^‡^ | 1.63 | 0.48 | 0.50 | 1.47 | 2.36 | 1.75 |
|  | Percent increase^§^ | 27.19 | 9.09 | 10.53 | 25.77 | 26.77 | 32.82 |
| Lung/bronchus cancer | E | 0.67 | -^¶^ | - | 0.45 | 1.05 | 0.86 |
|  | E+H | 0.91 | - | - | 0.58 | 1.44 | 1.20 |
|  | Percent increase | 36.71 | - | - | 29.73 | 36.61 | 40.00 |
| Other primary cancer | E | 3.06 | 0.57 | 1.05 | 2.51 | 3.87 | 4.13 |
|  | E+H | 4.76 | 0.69 | 1.58 | 3.61 | 6.07 | 6.69 |
|  | Percent increase | 55.66 | 21.43 | 50.00 | 44.23 | 56.93 | 61.95 |
| Metastatic cancer | E | 1.77 | 0.32 | 0.64 | 1.67 | 2.48 | 1.94 |
|  | E+H | 2.30 | 0.44 | 0.81 | 2.26 | 3.29 | 2.35 |
|  | Percent increase | 29.52 | 37.50 | 25.93 | 35.25 | 32.58 | 21.35 |
| Chronic obstructive pulmonary disease | E | 6.73 | - | 0.64 | 4.29 | 9.90 | 9.58 |
|  | E+H | 10.50 | 0.36 | 1.34 | 6.79 | 14.66 | 15.55 |
|  | Percent increase | 56.07 | - | 107.41 | 58.43 | 48.05 | 62.32 |
|  | CCDSS^¥^ |  |  | 2.43 | 9.78 | 17.98 | 26.20 |
| Other chronic lower respiratory disease | E | 4.46 | 1.66 | 3.56 | 4.52 | 5.44 | 4.46 |
|  | E+H | 11.35 | 4.40 | 8.00 | 10.56 | 13.88 | 12.45 |
|  | Percent increase | 154.38 | 165.85 | 124.83 | 133.87 | 155.36 | 179.41 |
| Asthma | E | 1.81 | 3.47 | 3.37 | 2.25 | 1.52 | 0.67 |
|  | E+H | 3.68 | 4.64 | 4.70 | 4.01 | 3.58 | 2.83 |
|  | Percent increase | 103.58 | 33.72 | 39.72 | 78.07 | 135.19 | 325.76 |
|  | CCDSS |  | 14.52 | 9.35 | 9.72 | 10.49 | 11.38 |
| Cystic fibrosis | E | - | - | 0.00 | 0.00 | 0.00 | - |
|  | E+H | 0.03 | - | - | - | - | - |
|  | Percent increase | - | - | - | - | - | - |
| Diabetes mellitus | E | 35.03 | 10.38 | 22.70 | 36.72 | 45.39 | 33.86 |
|  | E+H | 37.84 | 11.19 | 24.30 | 38.44 | 48.52 | 38.24 |
|  | Percent increase | 8.01 | 7.78 | 7.05 | 4.69 | 6.90 | 12.92 |
|  | CCDSS |  | 1.17 | 4.80 | 13.20 | 25.51 | 29.67 |
| Hypertension | E | 22.09 | 2.06 | 9.50 | 19.44 | 26.87 | 29.51 |
|  | E+H | 41.90 | 4.52 | 16.21 | 32.41 | 50.40 | 60.91 |
|  | Percent increase | 89.66 | 119.61 | 70.60 | 66.73 | 87.57 | 106.42 |
|  | CCDSS |  | 1.50 | 10.00 | 30.60 | 59.80 | 81.10 |
| Ischemic heart disease | E | 5.10 | 0.28 | 1.31 | 3.75 | 6.49 | 7.53 |
|  | E+H | 15.25 | 0.57 | 2.86 | 9.70 | 19.96 | 23.76 |
|  | Percent increase | 199.34 | 100.00 | 118.18 | 158.84 | 207.54 | 215.53 |
|  | CCDSS |  | 0.20 | 1.60 | 8.00 | 22.40 | 39.20 |
| Heart failure | E | 6.49 | 0.97 | 1.50 | 3.26 | 6.75 | 12.39 |
|  | E+H | 13.28 | 1.41 | 3.13 | 6.78 | 14.72 | 24.43 |
|  | Percent increase | 104.64 | 45.83 | 107.94 | 107.75 | 117.97 | 97.15 |
|  | CCDSS |  |  |  | 1.46 | 5.77 | 18.65 |
| Other heart disease | E | 8.98 | 2.18 | 2.36 | 5.02 | 10.20 | 15.48 |
|  | E+H | 19.45 | 4.04 | 5.16 | 9.91 | 21.34 | 35.28 |
|  | Percent increase | 116.59 | 85.19 | 118.18 | 97.36 | 109.32 | 127.95 |
| Stroke | E | 1.54 | 0.24 | 0.91 | 1.32 | 1.76 | 2.09 |
|  | E+H | 7.18 | 0.61 | 1.74 | 4.40 | 8.45 | 12.08 |
|  | Percent increase | 365.15 | 150.00 | 92.11 | 231.82 | 380.21 | 478.74 |
|  | CCDSS |  | 0.13 | 0.61 | 2.24 | 6.79 | 17.15 |
| Chronic kidney disease | E | 11.22 | 2.02 | 4.97 | 8.12 | 13.88 | 15.90 |
|  | E+H | 15.23 | 2.75 | 6.18 | 9.88 | 18.35 | 23.30 |
|  | Percent increase | 35.73 | 36.00 | 24.52 | 21.66 | 32.18 | 46.54 |
| Chronic liver disease | E | 1.61 | 1.66 | 2.08 | 2.30 | 1.78 | 0.64 |
|  | E+H | 2.92 | 2.46 | 3.51 | 4.08 | 3.28 | 1.43 |
|  | Percent increase | 81.79 | 48.78 | 68.97 | 77.49 | 84.66 | 125.40 |
| Schizophrenia | E | 0.81 | 1.70 | 1.22 | 1.01 | 0.89 | 0.15 |
|  | E+H | 1.67 | 2.83 | 2.27 | 1.99 | 1.99 | 0.51 |
|  | Percent increase | 106.62 | 66.67 | 86.27 | 96.43 | 123.16 | 240.00 |
|  | CCDSS |  | 0.82 | 1.14 | 1.29 | 1.06 | 0.80 |
| Dementia | E | 8.19 | 0.00 | - | 1.28 | 7.09 | 20.64 |
|  | E+H | 11.43 | - | - | 1.81 | 9.96 | 28.64 |
|  | Percent increase | 39.65 | - | - | 41.51 | 40.45 | 38.79 |
|  | CCDSS |  |  |  |  | 2.43 | 18.98 |
| Epilepsy | E | 0.53 | 0.81 | 0.67 | 0.72 | 0.57 | 0.19 |
|  | E+H | 1.87 | 2.10 | 1.86 | 2.53 | 2.10 | 1.02 |
|  | Percent increase | 253.19 | 160.00 | 178.57 | 250.00 | 265.57 | 431.58 |
|  | CCDSS |  | 0.87 | 0.85 | 1.04 | 1.09 | 1.19 |
| Multiple sclerosis | E | 0.24 | - | 0.29 | 0.45 | 0.23 | 0.09 |
|  | E+H | 0.41 | - | - | 0.63 | 0.43 | 0.24 |
|  | Percent increase | 67.44 | - | - | 40.54 | 91.67 | 166.67 |
|  | CCDSS |  | 0.09 | 0.30 | 0.42 | 0.34 | 0.14 |
| Parkinsonism | E | 1.12 | 0.00 | - | 0.41 | 1.59 | 1.96 |
|  | E+H | 1.87 | - | - | 0.60 | 2.58 | 3.41 |
|  | Percent increase | 67.09 | - | - | 47.06 | 62.13 | 74.23 |
|  | CCDSS |  |  |  | 0.17 | 0.90 | 2.10 |
| Other nervous system disorder | E | 4.14 | 2.95 | 4.37 | 4.89 | 4.72 | 3.08 |
|  | E+H | 12.35 | 6.99 | 9.19 | 12.08 | 15.06 | 12.34 |
|  | Percent increase | 198.37 | 136.99 | 110.38 | 147.04 | 218.92 | 300.00 |
| Rheumatoid arthritis | E | 0.00 | 0.00 | 0.00 | 0.00 | 0.00 | 0.00 |
|  | E+H | 0.66 | - | - | 0.49 | 1.00 | 0.76 |
|  | Percent increase | undefined | - | undefined | undefined | undefined | undefined |
|  | CCDSS |  |  | 0.58 | 1.51 | 2.91 | 4.27 |
| Other inflammatory rheumatic disease | E | 0.35 | 0.24 | 0.55 | 0.54 | 0.31 | 0.18 |
|  | E+H | 1.07 | 0.53 | 1.07 | 1.11 | 1.08 | 1.16 |
|  | Percent increase | 204.00 | 116.67 | 95.65 | 104.44 | 248.48 | 538.89 |
| Immune deficiency | E | 0.30 | 0.36 | 0.50 | 0.48 | 0.31 | - |
|  | E+H | 0.68 | 0.57 | 1.43 | 1.01 | 0.66 | 0.15 |
|  | Percent increase | 129.25 | 55.56 | 185.71 | 110.00 | 112.12 | - |
| Thalassemia | E | 0.08 | 0.20 | - | 0.14 | - | - |
|  | E+H | 0.17 | 0.40 | 0.17 | 0.20 | 0.12 | 0.13 |
|  | Percent increase | 114.29 | 100.00 | - | 41.67 | - | - |
| Sickle cell disorders | E | 0.09 | 0.44 | 0.29 | 0.06 | - | - |
|  | E+H | 0.14 | 0.57 | 0.45 | - | 0.08 | - |
|  | Percent increase | 59.38 | 27.27 | 58.33 | - | - | - |
| Down syndrome | E | 0.16 | 0.44 | 0.29 | 0.34 | 0.05 | 0.00 |
|  | E+H | 0.22 | 0.48 | 0.33 | 0.49 | 0.09 | 0.00 |
|  | Percent increase | 37.50 | 9.09 | 16.67 | 46.43 | 100.00 | undefined |
| Transplant recipient | E | 0.67 | 0.69 | 0.74 | 1.08 | 0.87 | 0.09 |
|  | E+H | 1.11 | 0.89 | 1.19 | 1.71 | 1.46 | 0.24 |
|  | Percent increase | 64.44 | 29.41 | 61.29 | 57.78 | 68.48 | 166.67 |
| Obesity | E | 2.21 | 3.15 | 3.32 | 3.05 | 2.45 | 0.54 |
|  | E+H | 3.46 | 4.81 | 4.58 | 4.35 | 4.16 | 1.15 |
|  | Percent increase | 56.63 | 52.56 | 38.13 | 42.69 | 70.00 | 111.11 |
|  | Statistics Canada^£^ |  | 21.0 | 31.7 | 32.6 | 28.3 |  |
| Note: Blank cells indicate estimates are not available. E = episode; E+H = episode plus history; CCDSS = Canadian Chronic Disease Surveillance System. *Number of Canadians with a confirmed or suspected COVID-19 diagnosis recorded in an acute care hospitalization in the Discharge Abstract Database for fiscal years 2019-2020 and 2020-2021. Acute care hospitalizations occurring in Quebec are not included.  ϮPrevalence of chronic condition (%) using diagnoses in the COVID-19 episode of care. Conditions arising during the COVID-19 episode of care are not included.  ‡Prevalence of chronic condition (%) using diagnoses in the COVID-19 episode of care and previous 10 fiscal years of acute care hospitalizations, except for cancers and obesity for which the previous two fiscal years are used. Conditions arising during the COVID-19 episode of care are not included. §Percent increase is calculated as follows: (E+H - E)/E * 100. ¶For confidentiality, estimates based on 1 to 4 people having the chronic condition are suppressed. Additional estimates may be suppressed to prevent residual disclosure through differencing across tables. ¥The most recent prevalence estimates (fiscal year 2016) were extracted from the Canadian Chronic Disease Surveillance System data tool.^1^ £Estimates are for calendar year 2020. Estimates for ages 20 to 34 and 65 to 79 years are based on data for Canadians age 18 to 34 and 65+ years, respectively. Obesity was defined as self-reported body mass index of 30 kg/m^2^ or greater.^2^ | | | | | | | |
| References:  1. Government of Canada [Internet]. Canadian Chronic Disease Surveillance System (CCDSS) [cited 2021 Jul 30]. Available from: https://health-infobase.canada.ca/ccdss/.   2. Statistics Canada [Internet]. Table 13-10-0096-01 Health characteristics, annual estimates [cited 2021 Oct 12]. doi: https://doi.org/10.25318/1310009601-eng. | | | | | | | |
