# Supplementary material for: Associations between chronic conditions and death in hospital among adults (aged 20+ years) during first acute care hospitalizations with a confirmed or suspected COVID-19 diagnosis in Canada
Source: PLoS One. 2023 Jan 4;18(1):e0280050. doi: 10.1371/journal.pone.0280050 (PMC9812329; doi:10.1371/journal.pone.0280050)
Supplement: S6 Table — (DOCX) [file pone.0280050.s006.docx]

| S6 Table. Prevalence of chronic conditions among adults aged 80+ years during first acute care hospitalizations with a confirmed or suspected COVID-19 diagnosis in Canada by period of admission | | | | | |
| --- | --- | --- | --- | --- | --- |
| Chronic Conditions | Admitted prior to  Sep 1, 2020  N*=2146 | | Admitted Sep 1, 2020 or later N=7773 | | Fisher’s exact  p-value  (two-sided) |
|  | N^Ϯ^ | Percent^‡^ | N | Percent |  |
| hematopoietic/lymphoid cancer | 41 | 1.9 | 133 | 1.7 | 0.5166 |
| lung/bronchus cancer | 23 | 1.1 | 96 | 1.2 | 0.6539 |
| other primary cancer | 132 | 6.2 | 532 | 6.8 | 0.2622 |
| metastatic cancer | 52 | 2.4 | 181 | 2.3 | 0.8092 |
| chronic obstructive pulmonary disease | 315 | 14.7 | 1227 | 15.8 | 0.2133 |
| other chronic lower respiratory disease | 288 | 13.4 | 947 | 12.2 | 0.1299 |
| asthma | 67 | 3.1 | 214 | 2.8 | 0.3777 |
| cystic fibrosis | 0 | 0.0 | -^¶^ | - | - |
| diabetes mellitus | 819 | 38.2 | 2974 | 38.3 | 0.9400 |
| hypertension | 1364 | 63.6 | 4678 | 60.2 | 0.0047 |
| ischemic heart disease | 503 | 23.4 | 1854 | 23.9 | 0.7096 |
| heart failure | 513 | 23.9 | 1910 | 24.6 | 0.5325 |
| other heart disease | 782 | 36.4 | 2717 | 35.0 | 0.2021 |
| stroke | 265 | 12.4 | 933 | 12.0 | 0.6536 |
| chronic kidney disease | 472 | 22.0 | 1839 | 23.7 | 0.1126 |
| chronic liver disease | 29 | 1.4 | 113 | 1.5 | 0.8373 |
| schizophrenia | 14 | 0.7 | 37 | 0.5 | 0.3080 |
| dementia | 708 | 33.0 | 2133 | 27.4 | <0.0001 |
| epilepsy | 27 | 1.3 | 74 | 1.0 | 0.2241 |
| multiple sclerosis | 7 | 0.3 | 17 | 0.2 | 0.3318 |
| parkinsonism | 83 | 3.9 | 255 | 3.3 | 0.2013 |
| other nervous system disorder | 263 | 12.3 | 961 | 12.4 | 0.9115 |
| rheumatoid arthritis | 15 | 0.7 | 60 | 0.8 | 0.8880 |
| other inflammatory rheumatic disease | 26 | 1.2 | 89 | 1.1 | 0.8197 |
| immune deficiency | - | - | - | - | - |
| thalassemia | - | - | - | - | - |
| sickle cell disorders | 0 | 0.0 | - | - | - |
| Down syndrome | 0 | 0.0 | 0 | 0.0 | undefined |
| transplant recipient | 7 | 0.3 | 17 | 0.2 | 0.3318 |
| obesity | 18 | 0.8 | 96 | 1.2 | 0.1377 |
| Note: Includes acute care hospitalizations ending by March 31, 2021 in Canada, excluding Quebec. Sep 1, 2020 was used to categorize patients into the first or second waves of the pandemic. COVID-19 = coronavirus disease 2019.  *Number of individuals in period of admission group.  ϮNumber of individuals with chronic condition.  ‡Percentage of individuals with chronic condition.  ¶For confidentiality, estimates based on 1 to 4 people having a chronic condition are suppressed, and additional estimates may be suppressed to prevent residual disclosure through differencing across tables. | | | | | |
