# Supplementary material for: Associations between chronic conditions and death in hospital among adults (aged 20+ years) during first acute care hospitalizations with a confirmed or suspected COVID-19 diagnosis in Canada
Source: PLoS One. 2023 Jan 4;18(1):e0280050. doi: 10.1371/journal.pone.0280050 (PMC9812329; doi:10.1371/journal.pone.0280050)
Supplement: S7 Table — (DOCX) [file pone.0280050.s007.docx]

| S7 Table. Prevalence of chronic conditions among females aged 20 to 49 years during first acute care hospitalizations with a confirmed or suspected COVID-19 diagnosis in Canada by pregnancy status | | | | | |
| --- | --- | --- | --- | --- | --- |
| Chronic Conditions | Not pregnant  N*=2480 | | Pregnant N=869 | | Fisher’s exact  p-value  (two-sided) |
|  | N^Ϯ^ | Percent^‡^ | N | Percent |  |
| hematopoietic/lymphoid cancer | 16 | 0.6 | 0 | 0.0 | 0.0176 |
| lung/bronchus cancer | -^¶^ | - | 0 | 0.0 | - |
| other primary cancer | 53 | 2.1 | - | - | - |
| metastatic cancer | 33 | 1.3 | 0 | 0.0 | <0.0001 |
| chronic obstructive pulmonary disease | 31 | 1.3 | - | - | - |
| other chronic lower respiratory disease | 163 | 6.6 | 7 | 0.8 | <0.0001 |
| asthma | 177 | 7.1 | 11 | 1.3 | <0.0001 |
| cystic fibrosis | - | - | 0 | 0.0 | - |
| diabetes mellitus | 600 | 24.2 | 16 | 1.8 | <0.0001 |
| hypertension | 300 | 12.1 | - | - | - |
| ischemic heart disease | 43 | 1.7 | 0 | 0.0 | <0.0001 |
| heart failure | 58 | 2.3 | - | - | - |
| other heart disease | 116 | 4.7 | 8 | 0.9 | <0.0001 |
| stroke | 35 | 1.4 | 0 | 0.0 | <0.0001 |
| chronic kidney disease | 136 | 5.5 | - | - | - |
| chronic liver disease | 98 | 4.0 | - | - | - |
| schizophrenia | 56 | 2.3 | - | - | - |
| dementia | - | - | 0 | 0.0 | - |
| epilepsy | 52 | 2.1 | 5 | 0.6 | 0.0020 |
| multiple sclerosis | 10 | 0.4 | 0 | 0.0 | 0.0729 |
| parkinsonism | 0 | 0.0 | 0 | 0.0 | undefined |
| other nervous system disorder | 278 | 11.2 | 10 | 1.2 | <0.0001 |
| rheumatoid arthritis | 7 | 0.3 | 0 | 0.0 | 0.2013 |
| other inflammatory rheumatic disease | 35 | 1.4 | 0 | 0.0 | <0.0001 |
| immune deficiency | 36 | 1.5 | 0 | 0.0 | <0.0001 |
| thalassemia | 7 | 0.3 | 7 | 0.8 | 0.0606 |
| sickle cell disorders | 17 | 0.7 | - | - | - |
| Down syndrome | 12 | 0.5 | 0 | 0.0 | 0.0444 |
| transplant recipient | 27 | 1.1 | 0 | 0.0 | 0.0005 |
| obesity | 145 | 5.8 | 41 | 4.7 | 0.2287 |
| Note: Includes acute care hospitalizations ending by March 31, 2021 in Canada, excluding Quebec. COVID-19 = coronavirus disease 2019.  *Number of individuals in pregnancy status group.  ϮNumber of individuals with chronic condition.  ‡Percentage of individuals with chronic condition.  ¶For confidentiality, estimates based on 1 to 4 people having a chronic condition are suppressed. | | | | | |
