# Supplementary material for: Associations between chronic conditions and death in hospital among adults (aged 20+ years) during first acute care hospitalizations with a confirmed or suspected COVID-19 diagnosis in Canada
Source: PLoS One. 2023 Jan 4;18(1):e0280050. doi: 10.1371/journal.pone.0280050 (PMC9812329; doi:10.1371/journal.pone.0280050)
Supplement: S8 Table — (DOCX) [file pone.0280050.s008.docx]

| S8 Table. Prevalence of chronic conditions among adults aged 20 to 79 years during first acute care hospitalizations with a confirmed or suspected COVID-19 diagnosis in Canada by Down syndrome status | | | | | |
| --- | --- | --- | --- | --- | --- |
| Chronic Conditions | No Down syndrome  N*=25523 | | Down  syndrome N=77 | | Fisher’s exact  p-value (two-sided) |
|  | N^Ϯ^ | Percent^‡^ | N | Percent |  |
| hematopoietic/lymphoid cancer | 406 | 1.6 | 0 | 0.0 | 0.6372 |
| lung/bronchus cancer | 205 | 0.8 | 0 | 0.0 | 1.0000 |
| other primary cancer | 1028 | 4.0 | 0 | 0.0 | 0.0773 |
| metastatic cancer | 583 | 2.3 | 0 | 0.0 | 0.4253 |
| chronic obstructive pulmonary disease | 2186 | 8.6 | -^¶^ | - | - |
| other chronic lower respiratory disease | 2781 | 10.9 | 16 | 20.8 | 0.0097 |
| asthma | 1024 | 4.0 | - | - | - |
| cystic fibrosis | 11 | 0.0 | 0 | 0.0 | 1.0000 |
| diabetes mellitus | 9630 | 37.7 | 16 | 20.8 | 0.0020 |
| hypertension | 8830 | 34.6 | 11 | 14.3 | <0.0001 |
| ischemic heart disease | 3061 | 12.0 | 0 | 0.0 | 0.0001 |
| heart failure | 2287 | 9.0 | 7 | 9.1 | 0.8434 |
| other heart disease | 3402 | 13.33 | 6 | 7.8 | 0.1797 |
| stroke | 1350 | 5.3 | - | - | - |
| chronic kidney disease | 3091 | 12.1 | 7 | 9.1 | 0.5978 |
| chronic liver disease | 894 | 3.5 | - | - | - |
| schizophrenia | 540 | 2.1 | - | - | - |
| dementia | 1196 | 4.7 | 24 | 31.2 | <0.0001 |
| epilepsy | 555 | 2.2 | 8 | 10.4 | 0.0003 |
| multiple sclerosis | 120 | 0.5 | 0 | 0.0 | 1.0000 |
| parkinsonism | 327 | 1.3 | 0 | 0.0 | 1.0000 |
| other nervous system disorder | 3150 | 12.3 | 12 | 15.6 | 0.3844 |
| rheumatoid arthritis | 159 | 0.6 | 0 | 0.0 | 1.0000 |
| other inflammatory rheumatic disease | 265 | 1.0 | 0 | 0.0 | 1.0000 |
| immune deficiency | 227 | 0.9 | - | - | - |
| thalassemia | 47 | 0.2 | 0 | 0.0 | 1.0000 |
| sickle cell disorders | 47 | 0.2 | 0 | 0.0 | 1.0000 |
| transplant recipient | 368 | 1.4 | - | - | - |
| obesity | 1112 | 4.4 | - | - | - |
| Note: Includes acute care hospitalizations ending by March 31, 2021 in Canada, excluding Quebec. Analysis was limited to age groups containing people with Down syndrome. COVID-19 = coronavirus disease 2019.  *Number of individuals in Down syndrome status group.  ϮNumber of individuals with chronic condition.  ‡Percentage of individuals with chronic condition.  ¶For confidentiality, estimates based on 1 to 4 people having a chronic condition are suppressed. | | | | | |
