# Supplementary material for: Associations between chronic conditions and death in hospital among adults (aged 20+ years) during first acute care hospitalizations with a confirmed or suspected COVID-19 diagnosis in Canada
Source: PLoS One. 2023 Jan 4;18(1):e0280050. doi: 10.1371/journal.pone.0280050 (PMC9812329; doi:10.1371/journal.pone.0280050)
Supplement: S9 Table — (DOCX) [file pone.0280050.s009.docx]

| S9 Table. Association between Charlson comorbidity index and death in hospital among adults aged 20+ years during first acute care hospitalizations with a confirmed or suspected COVID-19 diagnosis in Canada by life-course age group | | | | | |
| --- | --- | --- | --- | --- | --- |
| Charlson comorbidity  index | 20 to 34 (D=53/N=2476) | 35 to 49 (D=161/N=4189) | 50 to 64 (D=822/N=8303) | 65 to 79 (D=2509/N=10632) | 80+ (D=3890/N=9919) |
|  | aOR (95% CI) | aOR (95% CI) | aOR (95% CI) | aOR (95% CI) | aOR (95% CI) |
| 1 vs 0 | -* | 2.59 (1.56,4.18) | 2.96 (2.40,3.63) | 1.84 (1.62,2.10) | 1.23 (1.08,1.42) |
| 2 vs 0 | 3.90 (1.52,8.82) | 4.72 (2.93,7.45) | 3.63 (2.86,4.58) | 1.94 (1.68,2.25) | 1.46 (1.29,1.65) |
| 3+ vs 0 | 19.49 (9.95,37.74) | 9.33 (6.26,13.86) | 5.40 (4.13,7.03) | 2.54 (2.18,2.96) | 1.57 (1.38,1.79) |
| 4 vs 0 |  |  | 5.71 (4.14,7.77) | 2.80 (2.32,3.39) | 2.09 (1.79,2.45) |
| 5 vs 0 |  |  | 7.75 (5.17,11.43) | 3.38 (2.68,4.25) | 2.07 (1.71,2.51) |
| 6+ vs 0 |  |  | 8.61 (6.52,11.33) | 3.63 (3.04,4.34) | 2.41 (2.01,2.90) |
| Note: Includes acute care hospitalizations ending by March 31, 2021 in Canada, excluding Quebec. All final multivariable models are adjusted for sex and age, irrespective of statistical significance, and pregnancy status and period of admission when significant at an alpha of 0.05. For age groups 20 to 34 and 35 to 49 years, the highest category is a Charlson comorbidity index of 3 or greater; for all other age groups, the highest category is a Charlson comorbidity index of 6 or greater. For details on calculating the Charlson comorbididty index, see S3 Table. aOR = adjusted odds ratio, CI = confidence interval, COVID-19 = coronavirus disease 2019, D = in-hospital deaths in the age group, N = number of adults in the age group.  *For confidentiality, estimates based on 1 to 4 in-hospital deaths are suppressed. | | | | | |
