# Supplementary material for: Associations between chronic conditions and death in hospital among adults (aged 20+ years) during first acute care hospitalizations with a confirmed or suspected COVID-19 diagnosis in Canada
Source: PLoS One. 2023 Jan 4;18(1):e0280050. doi: 10.1371/journal.pone.0280050 (PMC9812329; doi:10.1371/journal.pone.0280050)
Supplement: S11 Table — (DOCX) [file pone.0280050.s011.docx]

| S11 Table. Prevalence of chronic conditions among adults aged 50 to 79 years during first acute care hospitalizations with a confirmed or suspected COVID-19 diagnosis in Canada by COVID-19 status | | | | | |
| --- | --- | --- | --- | --- | --- |
| Chronic Conditions | Confirmed  COVID-19  N*=18335 | | Suspected  COVID-19 N=600 | | Fisher’s exact  p-value (two-sided) |
|  | N^Ϯ^ | Percent^‡^ | N | Percent |  |
| hematopoietic/lymphoid cancer | 352 | 1.9 | 21 | 3.5 | 0.0104 |
| lung/bronchus cancer | 181 | 1.0 | 20 | 3.3 | <0.0001 |
| other primary cancer | 881 | 4.8 | 64 | 10.7 | <0.0001 |
| metastatic cancer | 500 | 2.7 | 38 | 6.3 | <0.0001 |
| chronic obstructive pulmonary disease | 2025 | 11.0 | 98 | 16.3 | 0.0001 |
| other chronic lower respiratory disease | 2255 | 12.3 | 98 | 16.3 | 0.0046 |
| asthma | 687 | 3.7 | 27 | 4.5 | 0.3266 |
| cystic fibrosis | -¶ | - | - | - | - |
| diabetes mellitus | 8138 | 44.4 | 213 | 35.5 | <0.0001 |
| hypertension | 7772 | 42.4 | 278 | 46.3 | 0.0589 |
| ischemic heart disease | 2807 | 15.3 | 120 | 20.0 | 0.0023 |
| heart failure | 2021 | 11.0 | 107 | 17.8 | <0.0001 |
| other heart disease | 2945 | 16.1 | 147 | 24.5 | <0.0001 |
| stroke | 1207 | 6.6 | 56 | 9.3 | 0.0123 |
| chronic kidney disease | 2687 | 14.7 | 84 | 14.0 | 0.7246 |
| chronic liver disease | 655 | 3.6 | 33 | 5.5 | 0.0191 |
| schizophrenia | 362 | 2.0 | 15 | 2.5 | 0.3700 |
| dementia | 1183 | 6.5 | 26 | 4.3 | 0.0339 |
| epilepsy | 412 | 2.2 | 21 | 3.5 | 0.0509 |
| multiple sclerosis | 90 | 0.5 | 8 | 1.3 | 0.0126 |
| parkinsonism | 314 | 1.7 | 10 | 1.7 | 1.0000 |
| other nervous system disorder | 2508 | 13.7 | 96 | 16.0 | 0.1041 |
| rheumatoid arthritis | - | - | - | - | - |
| other inflammatory rheumatic disease | 201 | 1.1 | 6 | 1.0 | 1.0000 |
| immune deficiency | 142 | 0.8 | 12 | 2.0 | 0.0037 |
| thalassemia | 30 | 0.2 | 0 | 0.0 | 1.0000 |
| sickle cell disorders | - | - | - | - | - |
| Down syndrome | - | - | - | - | - |
| transplant recipient | 286 | 1.6 | 11 | 1.8 | 0.6138 |
| obesity | 782 | 4.3 | 21 | 3.5 | 0.4104 |
| Note: Includes acute care hospitalizations ending by March 31, 2021 in Canada, excluding Quebec. COVID-19 = coronavirus disease 2019.  *Number of individuals in COVID-19 status group.  ϮNumber of individuals with chronic condition.  ‡Percentage of individuals with chronic condition.  ¶For confidentiality, estimates based on 1 to 4 people having a chronic condition are suppressed, and additional estimates may be suppressed to prevent residual disclosure through differencing across tables. | | | | | |
